# Coordinated interaction between Lon protease and catalase-peroxidase regulates virulence and oxidative stress management during Salmonellosis

**DOI:** 10.1080/19490976.2022.2064705

**Published:** 2022-04-19

**Authors:** Perumalraja Kirthika, Vijayakumar Jawalagatti, Amal Senevirathne, John Hwa Lee

**Affiliations:** a Department of Public Health, College of Veterinary Medicine, Jeonbuk National University, Republic of Korea; b Biochemistry & Molecular Biology Department, Mayo Clinic, Rochester, Minnesota, USA 55905; c Urology Department, Mayo Clinic, Rochester, Minnesota, USA 55905

**Keywords:** Proteome, Lon protease, KatG, ROS, macrophage survival, virulence

## Abstract

This study investigates the interplay between Lon protease and catalase-peroxidase (KatG) in relation to virulence modulation and the response to oxidative stress in *Salmonella* Typhimurium (ST). Proteomic comparison of ST wild-type and *lon* deletion mutant led to the recognition of a highly expressed KatG protein product among five other protein candidates that were significantly affected by *lon* deletion. By employing a bacterium two-hybrid assay (B2H), we demonstrated that the catalytic domain of Lon protease potentially interacts with the KatG protein that leads to proteolytic cleavage. Assessment of virulence gene expression in single and double *lon* and *katG* mutants revealed *katG* to be a potential positive modulator of both *Salmonella* pathogenicity Island-1 (SPI-1) and −2, while *lon* significantly affected SPI-1 genes. ST double deletion mutant, ∆*lon∆katG* was more susceptible to survival defects within macrophage-like cells and exhibited meager colonization of the mouse spleen compared to the single deletion mutants. The findings reveal a previously unknown function of Lon and KatG interaction in *Salmonella* virulence. Taken together, our experiments demonstrate the importance of Lon and KatG to cope with oxidative stress, for intracellular survival and *in vivo* virulence of *Salmonella*.

## Introduction

*Salmonella enterica* serovar Typhimurium (ST) is a facultative Gram-negative bacterium causing gastroenteritis and zoonotic infections^1^. During an infection, the sequential expression of *Salmonella* virulence genes modulates bacterial entry and colonization by circumventing the host immune system.^2,3^ During the process of infection, *Salmonella* senses various environmental stress conditions and responds accordingly to survive in the host environment.^4^ Elimination of various misfolded and faulty proteins is a vital physiological function for *Salmonella* survival. Additionally, the ability of the bacteria to sense and respond to environmental stress is important for their survival and replication in host cells.^4^ Under unfavorable environmental conditions, bacteria express proteins for the elimination of stress-damaged proteins. In bacteria, most intracellular proteolysis is initiated by members of four families of ATP-dependent proteases – including the Clp family (ClpAP and ClpXP), HslVU, FtsH and Lon.^5^ Among these, Lon is responsible for more than half of all the energy-dependent proteolysis in *Escherichia coli*,^6^ hence, similar functions of Lon can be expected in ST signifying its importance in virulence modulation.

The Lon protease plays a key role in *Salmonella* virulence, as it regulates the expression of virulence genes located in *Salmonella* pathogenicity Island I (SPI-1) during the early stages of systemic infection.^5^ It does not seem to have a crucial impact on SPI-2 genes, which are regulated during later phases of infection.^7^ Lon protease is shown to potentiate bacteria evolution and antimicrobial resistance.^8^ The dysregulation of Lon protease, a negative regulator of SPI-1 genes, leads to increased expression and orchestration of early virulence genes. Therefore, assessing the overexpressed proteins in the *lon-*deleted mutants may allow us to recognize key proteins that are regulated by *lon* and are crucial for bacterial survival and replication in the host. Previous studies have shown that the inactivation of *lon* leads to increased bacterial sensitivity to hydrogen peroxide (H_2_O_2_).^9^ Studies have shown that catalases; KatE, KatG, and KatN as well as alkyl hydroperoxide reductases; TsaA and AhpF play a vital role in combating the oxidative stress encountered by the bacteria in the host phagocytes.^10^ Among the catalases, KatE and KatG are heme catalases, whereas KatN is an Mn-catalase. The catalases scavenge endogenous and exogenous H_2_O_2_ and catalyze the decomposition of hydrogen peroxide to water and molecular oxygen.^11^ In ST, KatE and KatN are stationary-phase catalases that are induced by RpoS regulon^12,13^ whereas KatG is modulated by OxyR regulator.^10,14^ Furthermore, KatG has been reported to impart protection against aminoglycoside antibiotics and is essential for the oxidation of kanamycin antibiotic.^15^ The alkyl hydroperoxide reductases scavenge the H_2_O_2_ by directly converting it into water. Unlike other enteric bacteria, ST contains genes encoding both the alkyl hydroperoxide reductases; *tsaA* and *ahpF*. The catalytic efficiency of *tsaA/ahpC* was reported to be better than the aforementioned catalases thus it is crucial in combating the oxidative stress induced by lower levels of H_2_O_2._^16–18^ TsaA plays a vital role in protecting the gut microbes against toxicity and helps in the systemic dissemination of the infection.^19,20^

Although previous reports from our lab and others have shown that the deletion of *lon* increases the ST susceptibility to H_2_O_2_,^7,9^ the role of Lon protease in regulating the levels of crucial enzymes associated with H_2_O_2_ scavenging is yet to be elucidated. To study the effect of Lon protease on the whole *Salmonella* proteome with special emphasis on the H_2_O_2_ degrading enzymes, we conducted a proteome comparison of wild-type (WT) and *lon* mutant *Salmonella* strains intending to recognize differentially expressed proteins using 2D gel electrophoresis. The level of mRNA expressions does not directly represent the proteomic profile. However, direct proteomic assessment can be advantageous to elucidate differentially affected proteome.^21^ Once the candidate proteins are recognized, they can precisely be identified by mass spectrometry analysis or by sequencing.^22^ Here, we compare the proteomes of wild-type ST, JOL 401, to its *lon* inactivated derivative (JOL 909) grown under standard culture conditions. We aim to better understand the role of KatG in pathogenesis as well as its intersecting link with Lon protease if any. To this end, a double deletion mutant, ∆*katG*/∆*lon* was constructed, besides lack of catalase activity, this mutant will possess a global dysregulation of bacterial proteins. We hypothesized that the lack of both k*atG* and *lon* would lead to a severely impaired phenotype of ST. We, therefore, characterized the phenotypes of single deletion mutants, *Δlon* and *ΔkatG*, and a double deletion mutant, *Δlon/ΔkatG*, in comparison to the parental ST strain.

## Results

### *Deletion of* lon *influences the ST proteomic landscape*

**Figure 1.**
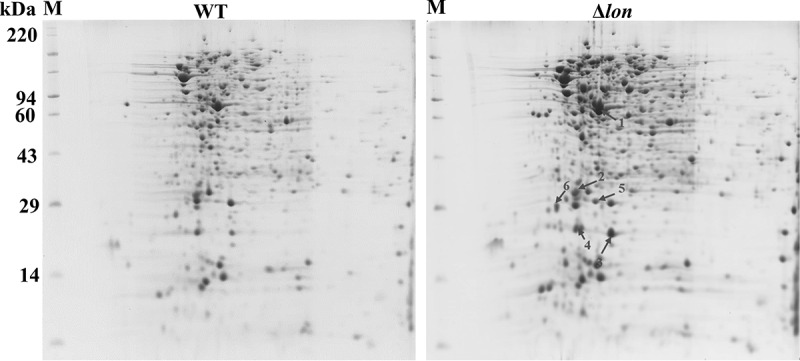
Proteomic comparison of *Salmonella* by 2-D gel electrophoresis. The whole proteomes were isolated from *Salmonella* Typhimurium wild-type (WT) and *lon* deletion mutant. Resolved gels were stained with Coomassie blue. Molecular standards represent myosin- 220 kDa; phosphorylase A- 94 kDa; catalase- 60 kDa; actin- 43 kDa; carbonic anhydrase- 29 kDa and lysozyme- 14 kDa. The selected differentially overexpressed protein spots are demarcated by numbers 1–6 (Table 1).

The effects of *lon* deletion on the ST proteomic landscape were analyzed using 2-D gel electrophoresis. The wild-type (WT) strain JOL 401 and its *lon* inactivated derivative JOL 909 were grown under identical conditions in LB broth until the late logarithmic phase of growth. Total proteins were extracted, resolved by 2-D gel electrophoresis, and stained with Coomassie blue (Figure 1). A comparison of the protein profile led to the identification of six protein spots that were more intense in the *lon* mutant than in the wild-type, suggesting that the genes encoding these proteins may be directly or indirectly regulated by *lon*. The six proteins were in abundance in *lon*-deleted mutants were designated using their molecular weights and are listed in Table 1. Then, the six prominent proteins were subjected to amino-terminal sequencing, and their sequence data were obtained. The amino acid sequences were compared against the NCBI database of *Salmonella enterica* serovar Typhimurium proteins (Table 1) for identification. Spots 1 and 2 were revealed to be catalase-peroxidase and manganese catalase, respectively. These are H_2_O_2_-dismutating enzymes that have a protective role against environmental H_2_O_2_ and are involved in stress adaptation.^23^ Spot 3 was alkyl peroxide reductase subunit C, a major protein in ST that protects bacteria from reactive nitrogen intermediates.^24^ Spot 4 was revealed to be OsmY, osmoprotectant import permease protein in ST. Spots 5 and 6 were found to be glucosamine-6-phosphate deaminase and D-ribose ABC transporter substrate-binding protein, respectively.

**Table 1.** Effect of the *lon* deletion on proteins expressed in *Salmonella* Typhimurium as observed by 2-D gel analysis of total proteins

Protein designation (kDa)	Expression in *Δlon*	N-terminal protein	Identical protein or homologue
79	Increased	MSTTDDTHNTLSTGKCPFHQ	Catalase/Peroxidase
31	Increased	MFRHVKQLQYTVRVSEPNPG	Manganese catalase
20	Increased	MSLINTKIKP FKNQAFKNGE	alkyl peroxide reductase subunit C
21	Increased	MTMTRLKISKTLLAVMLTSA	Molecular chaperon OsmY
29	Increased	MRLIPLSTAE QVGKWAARHI	glucosamine-6-phosphate deaminase
30	Increased	MNMKKLATLVSAVALSATVS	D-ribose ABC transporter substrate-binding protein

## KatG protein levels controlled by Lon protease

**Figure 2.**
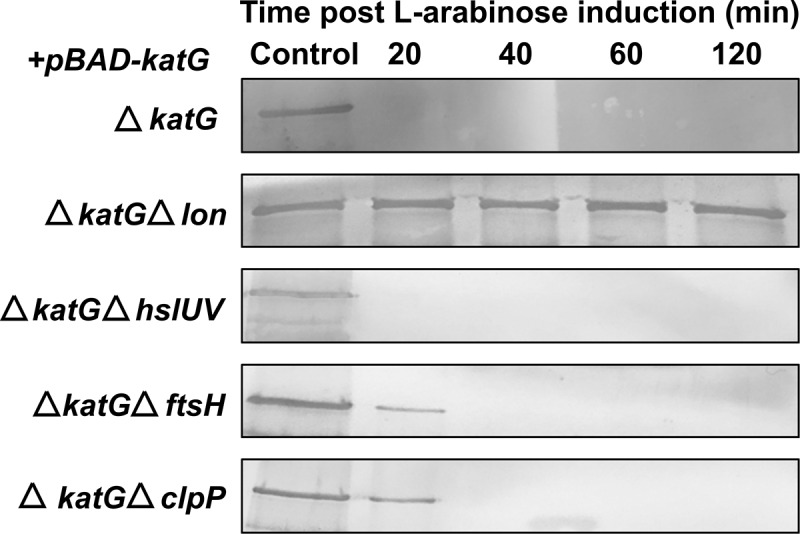
KatG proteolysis by Lon protease. *In vitro* assessment of plasmid-encoded KatG degradation in *Salmonella katG* mutants by western blot. Deletion mutants of *katG, katG lon, katG hslUV, katG ftsH*, and *katG clpP* were transformed with the pBAD-*katG* plasmid vector. Mid-log phase cultures were induced by L-arabinose (0.5 mM) under well-aerated conditions, and KatG expression was assessed in total proteins extracted from the transformants. KatG was probed by rabbit polyclonal antibodies. The level of KatG lysis is shown at 20-, 40-, 60-, and 120-min time intervals. Control lane- positive control from wildtype ST.

We hypothesized that one or more of the cytoplasmic proteases were responsible for the degradation of KatG in Gram-negative bacteria, including Lon protease.^25^ If the hypothesis is true, the absence of Lon protease will stabilize KatG in the bacterial cytoplasm. To test the aforementioned hypothesis, KatG was produced from the pBAD vector in a *katG*-deleted strain carrying an additional *lon, hslUV, ftsH*, or *clpP* gene deletion. After inhibition of protein synthesis using chloramphenicol, KatG stability was quantified by immunoblot. It was evident that KatG was efficiently degraded in all Lon protease intact *Salmonella* strains but was significantly stable in the *lon*-deleted mutant (Figure 2). The inactivation of *ftsH* and *clpP* increased KatG stability only partially. On the other hand, the deletion of *hslUV* did not affect KatG stability. These observations suggest that KatG is most likely degraded by the Lon protease.

## The proteolytic active site of Lon protease interacts with KatG

**Figure 3.**
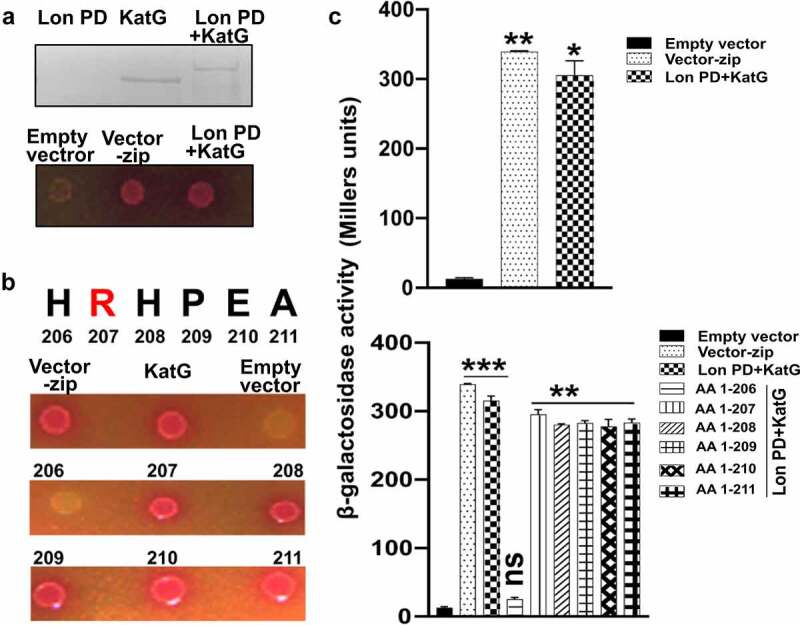
Confirmation of the KatG-Lon interaction. (a) The interaction between KatG and the Lon proteolytic domain (PD) was assessed by DSS cross-linking and bacteria two-hybrid (B2H) assay. Purified Lon and KatG were treated with DSS to make non-cleavable cross-linkers. Individual proteins and interacted proteins were resolved in SDS-PAGE and probed using KatG-specific rabbit polyclonal antibodies. The size difference between KatG and the KatG-Lon PD interaction (~99 kDa = 79 kDa + 20 kDa) represents a possible interaction. The same interaction was confirmed by the B2H assay. *Escherichia coli* BTH101 strains harboring two plasmids, pUT18 and pKT25 derivatives expressing a C-terminal fusion of the cyaA T18 fragment to the KatG coding region and N-terminal fusions of the cyaA T25 fragment to either the coding region of Lon PD or pKT25 empty vector (negative), were spotted onto a MacConkey-maltose plate and incubated at 30°C for 40 h. Red-colored colonies indicate a positive interaction. (b) Essential KatG domain prediction for Lon PD interaction by the B2H assay. The B2H assay was conducted using variable lengths of KatG C-terminus domains; C-terminal fusions of the cyaA T18 fragment to either the coding regions of the full-length *katG, katG1*-206 (206), *katG1*-207 (207), *katG1*-208 (208), *katG1*-209 (209), *katG1*-210 (210), or *katG1*-211 (211) genes or to the pUT18 empty vector were indicated. The pink-colored spots of cultures on MacConkey agar indicate potential interactions. (c) The interaction was validated by analyzing by β-galactosidase activity. The average β-galactosidase activities (β-Gal units) are shown in Miller Units. Empty-vector and vector-zip were negative and positive controls, respectively. Means were compared by Tukey’s multiple comparison test. Significant differences compared to the negative control are indicated (*p < .05, **p < .01, ***p < .001).

The Lon proteolytic domain (Lon PD) contains a conserved lysine (Lys722), located 43 residues beyond the catalytic serine (Ser679) to carry out catalytic activities.^26^ To understand how Lon protease is involved in the degradation of KatG, we investigated whether KatG physically interacts with the proteolytic active site of Lon protease. We used a bacterial two-hybrid assay to assess the interaction between KatG and the PD of Lon protease by expressing the *katG* gene with a C-terminal fusion of the *cyaA*-T18 fragment and *lon* PD genes with N-terminal fusions of the *cyaA*-T25 fragment in an *E. coli* strain lacking CyaA adenylate cyclase. We then spotted cells on a MacConkey agar plate containing maltose and measured β-galactosidase production from a cAMP-dependent promoter produced when T18 and T25 fragments of the *cyaA* gene are functionally complemented by a physical interaction between fused KatG and the PD of Lon protease. The strain expressing KatG-T18 and the T25-Lon proteolytic domain showed a strong red color on the MacConkey-maltose plate, indicating that KatG interacts with Lon PD (Figure 3a). By contrast, no interaction was observed in KatG-T18 co-expressing the empty T25 fragment. The interaction was confirmed by measuring the β-galactosidase activity. The KatG-Lon PD interaction was further analyzed by *in vitro* cross-linking of purified proteins. We observed an additional band corresponding to roughly 99 kDa (79 + 20) on polyacrylamide gel when KatG and the Lon proteolytic domain were incubated with a cross-linker (Figure 3a).

## The C-terminus domain beyond Arg207 of the KatG protein is essential for interaction with Lon PD

Given that the mechanism of Lon is similar to that of *E. coli* Type 1 signal peptidase (SPase) (MEROPS, clan SK; PDB codes: 1kn9, 1b12), we predicted KatG cleavage sites for Lon PD using PROSPER (PROtease substrate SPecificity servER) (Supplementary information). We then constructed a series of T18-fused *katG* derivatives deleted from their C-termini to determine the minimal requirement for Lon PD interaction. Interestingly, *katG* derivatives that harbored the coding region up to the amino acid 207 or more (up to 207, 208, 209, or 211) retained the ability to interact with Lon PD, but the derivatives with coding regions up to amino acid 206 or less (up to 206, 205, or 204) did not exhibit any interaction. These results indicate that the amino acid at position 207, which corresponds to arginine, in KatG is essential for interaction with Lon PD (Figure 3b,c).

## *katG and lon* deletion prevents *in vitro* ST survival

**Figure 4.**
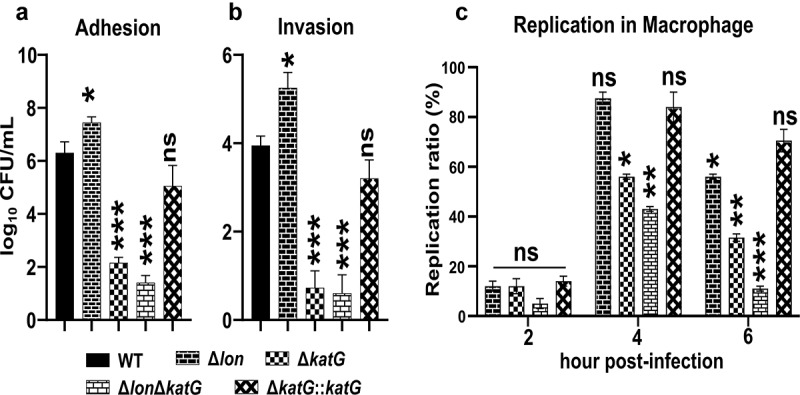
**Effect of KatG on *Salmonella* adhesion, invasion, and replication in macrophages**. (a) Adhesion and (b) Invasion of RAW264.7 cells were observed post-infection with WT, Δ*lon*, Δ*katG*, Δ*lonΔkatG*, and Δ*katG::katG* (MOI = 20). The data represent the adhesion and invasion (Log_10_ CFU/mL) ± the SD from at least three independent experiments performed in duplicates. (c) The replication ratio represents the intramacrophage survival of Δ*lon*, Δ*katG*, Δ*lonΔkatG*, and Δ*katG::katG* compared to that of the wild-type control at the indicated time points post-infection. Means were compared by Tukey’s multiple comparison test. Significant differences compared to the wild-type (WT) control are indicated (^ns^non-significant, *p < .05, **p < .01, ***p < .001).

The ability of ST to adhere, invade, and replicate inside macrophage cells is directly linked with virulence and systemic colonization of the host.^27^ We hypothesized that the inactivation of KatG might impact the ability of this mutant to invade and survive within macrophages. The deletion of only *lon* enhanced bacterial adhesion and invasion (Figure 4a,b). The adhesion and invasion assay revealed that the ∆*katG* mutants were associated and internalized into RAW cells less than the wild-type bacteria. Upon further comparison, the adhesion and invasion capacity of the bacteria was reduced in ∆*katG* mutants carrying an additional *lon* deletion.

Systemic dissemination is dependent on survival within phagocytic cells. To determine whether KatG contributes to intracellular replication, we assessed the intracellular replication of wild-type and mutants after 1, 2, and 3 h post-infection in RAW264.7 cells (Figure 4c). Compared to the wild-type, the ∆*katG* mutant exhibited defective intracellular persistence. Additionally, we observed a significant decrease in the replication of *∆lon* and ∆*katG*∆*lon* mutants compared to the wild-type in the macrophage cell line. These data support the hypothesis that KatG is needed for intracellular survival and growth in macrophages.

## Deletion of *katG* and *lon* enhances ST susceptibility to exogenous H_2_O_2_

**Figure 5.**
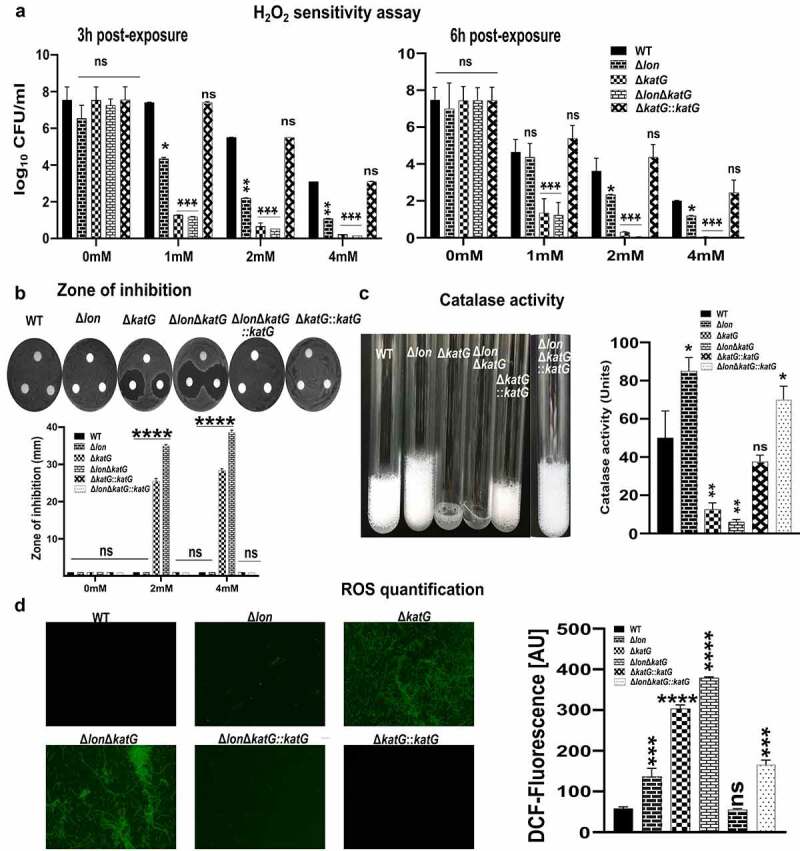
**H_2_O_2_ sensitivity, ROS production, and Catalase activity**. (a) Susceptibility of *Salmonella* strains WT, Δ*lon*, Δ*katG*, Δ*lonΔkatG*, and Δ*katG::katG* to oxidative stress was assessed following H_2_O_2_ exposure. An equal number of cells were exposed to 0, 1, 2, 4 mM H_2_O_2_ for 6 h, and the live cell numbers were determined by plating on agar. (b) Disk diffusion assay. A hundred microliters of each strain were spread on LB agar. Disks were saturated with 0, 2, and 4 mM and placed on bacterial lawns. Plates were incubated at 37°C for 12 h. Inhibition zones were compared. (c) Catalase activity of *Salmonella* strains was compared following exposure to 30% H_2_O_2_. An equal number of mid-log phase cultures were treated with 100 μl of 1% Triton X 100 and 30% H_2_O_2_. Foaming was compared after a 15-min reaction time. (d) Intracellular ROS assessment. The generation of ROS was assessed by DCF fluorescence assay. DCFH without cells was used as a negative control. DCF fluorescence intensities from WT, Δ*lon*, Δ*katG*, Δ*lonΔkatG*, Δ*katG::katG* and Δ*lonΔkatG::katG* were measured by a spectrofluorophotometer. Significant differences compared to the wild-type control are indicated (^ns^non-significant, *p < .05, **p < .01, ***p < .001, ****p < .0001).

Despite the reduced or complete absence of catalase activity, ∆*katG*, ∆*lon*, and ∆*katG*∆*lon* grew well in LB broth. To evaluate the adaptation of the bacterial strains to oxidative stress, we added H_2_O_2_, the substrate of the catalase, to the culture. The bacterial growth in the presence of 0, 1, 2, and 4 mM H_2_O_2_ was investigated. Compared to that of the wild-type, the growth of ∆*katG* and ∆*lon* was reduced at 2 mM and 4 mM H_2_O_2_, and the growth of ∆*katG*∆*lon* was further inhibited (Figure 5a). The deletion of stress regulating genes led to decreased bacterial survival upon exposure to H_2_O_2_ wherein each of the strains exhibited slightly different growth. This pattern was observed at 3 and 6 h post H_2_O_2_ exposure (Figure 5a). Additionally, the sensitivity of bacteria to H_2_O_2_ was indicated by the zone of inhibition. As shown in Figure 5b, the inhibitory zone diameters for ∆*katG* and ∆*lon* were bigger than that of the wild-type at each concentration of H_2_O_2_, while no differences were observed between the wild-type and the complementary strain. Further, the diameters of inhibitory zones for ∆*katG*∆*lon* were larger compared to those of the other strains (Figure 5b).

As the mutants were highly susceptible to H_2_O_2_ stress, we asked if the deletion has affected their catalase activity, the enzyme required to detoxify H_2_O_2_ to water and oxygen. Therefore, we monitored the catalase activity through the enzymatic decomposition of hydrogen peroxide. The oxygen generated during the reaction trapped by Triton X-100 was visualized as foam. The height of foam was higher in ∆*lon* compared to the wild-type which may be attributed to the accumulation of catalases, including KatG, in the bacteria devoid of Lon protease. While mutants ∆*katG* and *∆lon∆katG* did not exhibit any foam formation upon exposure to hydrogen peroxide (Figure 5c). We also evaluated the expression of *katE, katG, katN, tsaA*, and *ahpF* in the mutants. The expression levels of genes, such as *katE, katN*, and *tsaA* were downregulated in the *lon* mutant and an upregulated expression of *katG* and *ahpF* was observed. On the other hand, an increase in expression of *tsaA* and *ahpF* was noted in *katG* and double deletion *lon/katG* mutants (Supplementary Figure 1A).

## Absence of *katG* and *lon* causes the accumulation of intracellular ROS in ST

As the mutants exhibited high sensitivity to H_2_O_2_ with reduced or no detectable catalase activity, we studied the accumulation of intracellular ROS in wild-type and the mutants by fluorescent staining with DCFH-DA. The DCFH-DA is a non-fluorescent compound, however, upon oxidation by ROS forms a fluorescent product, 2’, 7’ – dichlorofluorescein (DCF) and the accumulation of DCF is widely applied to quantify the intracellular ROS.^28^ The ∆*katG* and ∆*lon* mutants exhibited a noticeably increased fluorescence compared to the wild-type bacteria (Figure 5d). The microscopic images of DCF fluorescence were higher in the double deletion mutant, ∆*katG*∆*lon*, compared to that of the single-gene deletion mutant (∆*katG)*. The meager DCF fluorescence in ∆*lon* mutants can be attributed to the accumulation of catalase enzyme. Furthermore, the DCF fluorescence was also measured by a spectrofluorophotometer to further validate the results obtained from fluorescence microscopy. The emission spectra also suggested maximum accumulation of ROS in ∆*katG*∆*lon* compared to the other ST strains (Figure 5d).

## *katG and lon* oppositely regulate the expression of SPI-2 not SPI-1 genes

**Figure 6.**
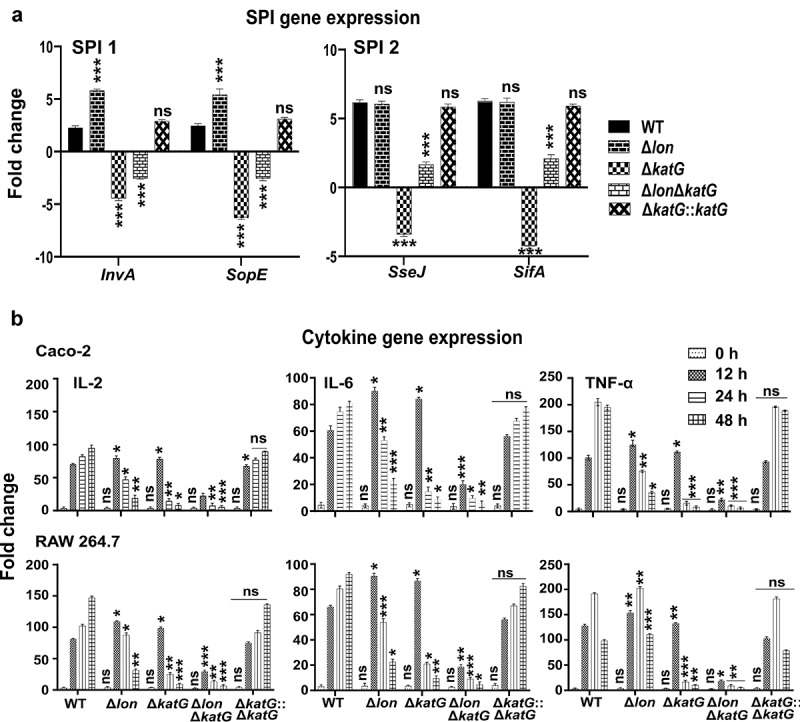
Gene expression analysis. (a) Transcriptional levels of SPI-1 and −2 effectors. The mRNA levels of SPI-1 genes *invA* and *sopE*, as well as SPI-2 genes *sseJ* and *sifA*, were evaluated as relative mean fold changes using *rrsG* as the endogenous control. (b) The mRNA levels of IL-2, IL-6, and TNF-α were recorded in WT, Δ*lon*, Δ*katG*, Δ*lonΔkatG*, and Δ*katG::katG*-infected Caco-2 and RAW264.7 cells at the indicated time points. The data were represented as relative mean fold change ± the SD from at least three independent experiments performed in duplicates. The experiment was performed using *GAPDH* as the endogenous control. Means were compared by Tukey’s multiple comparison test. Significant differences compared to the wild-type control are indicated (^ns^non-significant, *p < .05, **p < .01, ***p < .001). IL, interleukin; IFN, interferon; TNF, tumor necrosis factor.

Given that SPI-1 genes are upregulated during bacterial uptake by macrophages and SPI-2 genes are required for ST intra-macrophage survival,^29^ we studied the impact of *katG* deletion on SPI-1 and SPI-2 gene expression. The deletion of *lon* led to the upregulation of SPI-1 genes, whereas the mRNA levels of SPI-2 genes were insignificant compared to those of wild-type (Figure 6a). Inactivation of *katG* led to the downregulation of SPI-1 genes. The mRNA levels of *invA* and *sopE* were decreased by 4- and 6-folds in the adherent ∆*katG* mutant, respectively (Figure 6a). The expression of SPI-2 genes was studied in bacteria collected after 2 hours of infection. Our results showed that the transcriptional levels of *sseJ* and *sifA* were also downregulated in the absence of *katG*. The ∆*katG* mutant exhibited a 3-fold reduction in the expression levels of *sseJ* and a 4-fold decrease in *sifA* after 2 hours of infection (Figure 6a). The double deletion mutant ∆*katG*∆*lon* failed to upregulate both SPI-1 and −2 effector genes. Additionally, evaluation of the gene expression in bacteria grown in LB medium revealed a similar trend (Supplementary Figure 1B and 1C).

## Inactivation of *katG* and *lon* influences the host inflammatory response

Given that bacterial antioxidants harness H_2_O_2_ to restrict the host immune function,^30^ we hypothesized that the deletion of *katG* and *lon* could increase the secretion of host pro-inflammatory cytokines. To test this hypothesis, we infected RAW264.7 and Caco-2 cells with ST strains. The ∆*katG* mutant upregulated levels of pro-inflammatory cytokines up to 12 h post-infection in both the cell lines (Figure 6b). The levels of IL-2, IL-6, and TNF-α increased more than 80-fold upon infection with the ∆*katG* mutant. A similar observation was made for cells receiving the ∆*lon* mutant. On the other hand, there was a decrease in pro-inflammatory cytokine mRNA levels in cells receiving the ∆*katG* mutant 24 and 48 h post-infection. A reduction in the mRNA levels of pro-inflammatory cytokines was evident in cells infected with the ∆*lon* mutant (Figure 6b). The double deletion mutant ∆*katG*∆*lon* elicited a much lesser inflammatory response compared to that of ∆*katG* and ∆*lon* mutants wherein the host cells showed a <2-fold increase in the expression of pro-inflammatory cytokines.

## *katG and lon* help bacteria colonize the spleen after intraperitoneal infection

**Figure 7.**
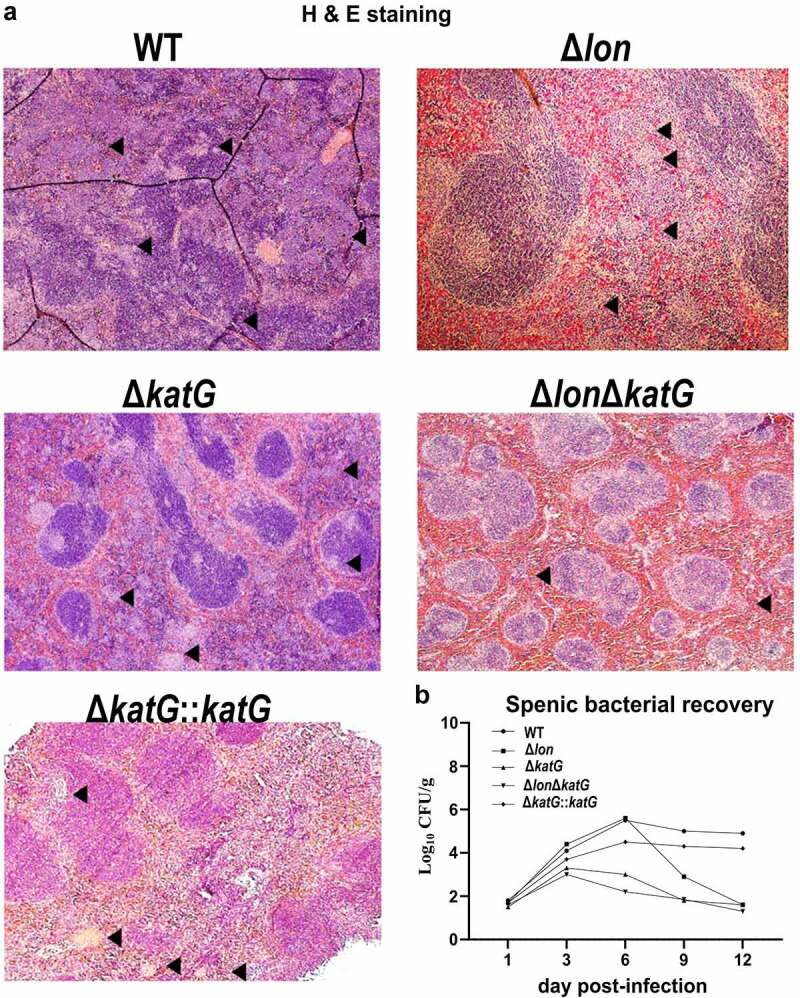
Histopathological analysis and splenic bacterial recovery. Pathological assessment was conducted on the mice models of ST infection (n = 10 per ST strain). Mice were inoculated with WT, Δ*lon*, Δ*katG*, Δ*lonΔkatG*, and Δ*katG::katG* at 1 × 10^5^ CFU/mouse/100 μl PBS intraperitoneally. At indicated day post-infection, mice were euthanized, and spleen samples were collected for (a) hematoxylin and eosin staining and (b) bacterial enumeration. Shown are the representative images of H & E staining of spleen collected at day 3 post-infection. Total spleens were harvested, homogenized, and used for bacterial counting. The data were represented as log_10_ CFU/g ± the SD from two independent experiments. Means were compared by Tukey’s multiple comparison test. Black arrowhead- damage to the pulp architecture.

Based on *in vitro* assays, we hypothesized that the inability of the ∆*katG* mutant to adhere and invade the macrophage-like cells could be linked to poor spleen colonization. In turn, we infected 10 *Salmonella*-susceptible BALB/c mice per ST strain intraperitoneally (i.p.). The deletion of *katG* led to poor colonization of the spleen, wherein <3log CFU/g of bacteria was recorded day 6 post-infection (Figure 7b). On the other hand, wild-type exhibited enhanced colonization, with >5log CFU/g. The *∆lon* and ∆*katG∆lon* mutants also exhibited lesser spleen colonizing abilities compared to the wild-type. The colonization-mediated tissue damage in the spleen was evaluated by H&E staining (Figure 7a). The ∆*katG* mutant caused minimum damage to the pulp architecture of the mice spleen; the wild-type caused damage associated with bacterial infection, including infiltration of inflammatory cells.

## Discussion

The objective of this study was to further our understanding of how the *Salmonella* proteome is affected by *lon* gene deletion. The inactivation of cytosolic Lon protease appears to impair the stress response mechanism and causes the accumulation of partially folded proteins related to *Salmonella* virulence.^31,32^ To address this possibility with respect to *lon* gene deletion, we used the combination of 2-D gel analysis of proteins to evaluate the global expression pattern. We compared the proteome of wild-type (WT) *Salmonella* to that of *lon* deletion mutant using 2-D gel electrophoresis. Due to high-resolution of protein separation, two proteomes could be compared to recognize differentially expressed proteins (Figure 1). The availability of a highly curated *Salmonella* genome is very helpful for the proteomic identification and characterization of candidate proteins essential for bacterial survival.^33^ The proteins overexpressed in the *lon* deletion mutant; spots 1, 2, 3, and 4, were recognized to be enzymes involved in regulating bacterial survival in oxidative and nitrosative stress conditions.^23,24^ Spot 5 corresponds to an enzyme involved in the metabolism of amino sugar^34^ whereas spot 6 represents the ABC transporter involved in the transport of ribose sugars essential for bacterial chemotaxis.^35^ These observations further confirm the role of Lon protease in *Salmonella* stress regulation and metabolism. Among five candidate proteins, spot 1, represented by the Catalase-peroxidase enzyme (KatG) and was selected for further studies due to the potential connection of this protein with oxidative stress management.^10,36^ The *katG* gene in ST encodes the KatG protein of approximately 79 kDa. We have established that Lon as a potential protease responsible for KatG degradation, whereas the other cytoplasmic proteases are unlikely to degrade this H_2_O_2_-dismutating enzyme (Figure 2). Additionally, this indicates that KatG most likely constitutes a substrate of the lon protease. For any proteolytic reaction, a physical interaction between the substrate and proteolytic domain is necessary.^37^ B2H assay and immunoprecipitation revealed that the Lon PD interacts with KatG (Figure 3a). Further B2H assays revealed that the amino acid arginine at position 271 of the KatG was crucial for its recognition by the Lon protease for proteolysis^38^ (Figure 3b).

To characterize KatG activity in relation to *Salmonella* virulence, we constructed both individual and double deletion mutants of *lon* and *katG* open reading frames. When the two proteins are intact, they coordinately regulate the response to intracellular oxidative stress and the expression of virulence genes, ensuring the systemic establishment of an infection.^7^ Here, we establish that Lon protease and KatG cooperate to regulate the virulence factors and are required for ST pathogenicity. The ability of ST to adhere, invade and proliferate inside the macrophages is associated with virulence and colonization of the host.^39,40^ The deletion of *katG* impaired the ability of the bacteria to adhere and invade macrophage-like cells, the double deletion mutant *∆lon∆katG* exhibited a further reduction in the adhesion and invasion abilities (Figure 4a,b). This followed by a diminished intramacrophage survival and replication of the *∆katG* and *∆lon∆katG* could be linked to the production of reactive oxygen species, ROS^27^ (Figure 4c). The ROS are formed intracellular as a by-product of normal aerobic metabolism in the bacteria^41^ and are capable of causing bacterial lysis if the accumulation of ROS is not controlled. Here, the accumulation of fluorescent DCF in the ∆*lon*, ∆*katG* and *∆lon∆katG* compared to the parental strain shows that the expression of these genes is required to mitigate the ROS-mediated damage^42^ and the deletion of *katG* and *lon* confers hypersensitivity to H_2_O_2_.^43,44^ Apart from the endogenous ROS, the phagocytic H_2_O_2_ that diffuses into the bacterial cytoplasm can kill the bacteria by damaging the DNA.^45^ Taking this into account we evaluated the ability of the ST wild-type and the mutants to resist the exogenously supplemented H_2_O_2_. The outcomes revealed that the inactivation of *lon* or *katG* restricted bacterial growth to a similar degree and completely blocked the growth of double deletion mutant, *∆lon∆katG* when exposed to H_2_O_2_ (Figure 5a). The overexpression of KatG in Lon mutants may be a compensatory mechanism to counteract the increased oxidative stress, where the KatG is activated as an attempting to degrade H_2_O_2_ to ensure survival of the mutant *Salmonella*. However, lon mutant with higher catalase activity had increased intracellular ROS that may be due to the dysregulated oxidative stress response. Earlier we have shown that the deletion of *lon* causes an increased accumulation of hydroxyl radicals in the *Salmonella*.^7^ In support, the expression levels of *katE, katN*, and *tsaA* were downregulated in *lon* mutant. It should be noted that the primary hydrogen peroxide scavenging role of *tsaA* (*ahpC*) over *katE, katN, katG*, and *ahpF* has been reported earlier.^46^ Therefore, intracellular ROS accumulation in *lon* mutant may be attributed to the dysregulated oxidative stress response, despite an increase in catalase activity. Further, increased oxidative stress cannot be fully counteracted by overexpressed catalase such as KatG due to the fact that H_2_O_2_ is highly detrimental to phagocytosed *Salmonella* especially for an attenuated one. Our results suggest that the collective coordination of Lon and catalases, in this case, KatG appeared to be essential during the early infection phase that is vital to establish a successful infection where both virulence regulation and timely responses against oxidative stress exerted by the host cell via an interplay between Lon and KatG proteins. ST injects an array of proteins into the macrophage cytoplasm *via* the SPI-1 and-2 T3SS system leading to invasion and intracellular replication. Our results show that *katG* regulates the SPI-1 genes that are involved in regulating the initial bacterial invasion of host cells^47^ as well as SPI-2 genes that are essential for intracellular survival.^48^ The downregulation of SPI-1 factors *invA* and *sopE* genes in the *∆katG* mutant shows the catalase-peroxidase enzyme influences the ST entry into the host cells (Figure 6a). The downregulation of SPI-2 effectors *sseJ* and *sifA* suggests that KatG of ST is involved in impairing the macrophage antimicrobial mechanism and assists bacterial replication intracellularly. Unlike Lon and PhoP/PhoQ regulatory system, KatG may modulate two major virulence pathways, invasion and survival in macrophages in the same direction for ST pathogenesis.

The dysregulation of bacterial virulence factors in the absence of *katG* may impact the host inflammatory response.^49^ The evaluation of the transcriptional levels of pro-inflammatory cytokines revealed that the mRNA expression of IL-2, IL-6 and TNF-α increased by ≥50 folds in RAW264.7 and Caco-2 cells receiving *∆katG* mutant 12 h post-infection. However, the levels of pro-inflammatory cytokines dropped to <10 folds at 24 h post-infection (Figure 6b). The initial upregulation of inflammatory response can be attributed to the presence of other H_2_O_2_ scavenging enzymes, as bacterial antioxidants are shown to use the host cellular H_2_O_2_ to modulate Ca^2+^ signaling and limit host immune function.^30^ The later reduction in the host response may be attributed to the rapid elimination of the *∆katG* mutant from the cells due to disruption in the virulence mechanism. Also, intramacrophage survival and replication are associated with the ST colonization of the mouse spleen.^9^ The *in vivo* colonization studies corroborated with the aforementioned finding wherein the *∆katG* mutant exhibited meager colonization in the mouse spleen from day 3 to 12 post-infection compared to the parental strain (Figure 7b). The inability of *∆katG* mutant to colonize the spleen led to minimal or no observable damage to the tissue architecture (Figure 7a) suggests that KatG plays a vital role in intramacrophage survival that is key to systemic infection in the host.

**Figure 8.**
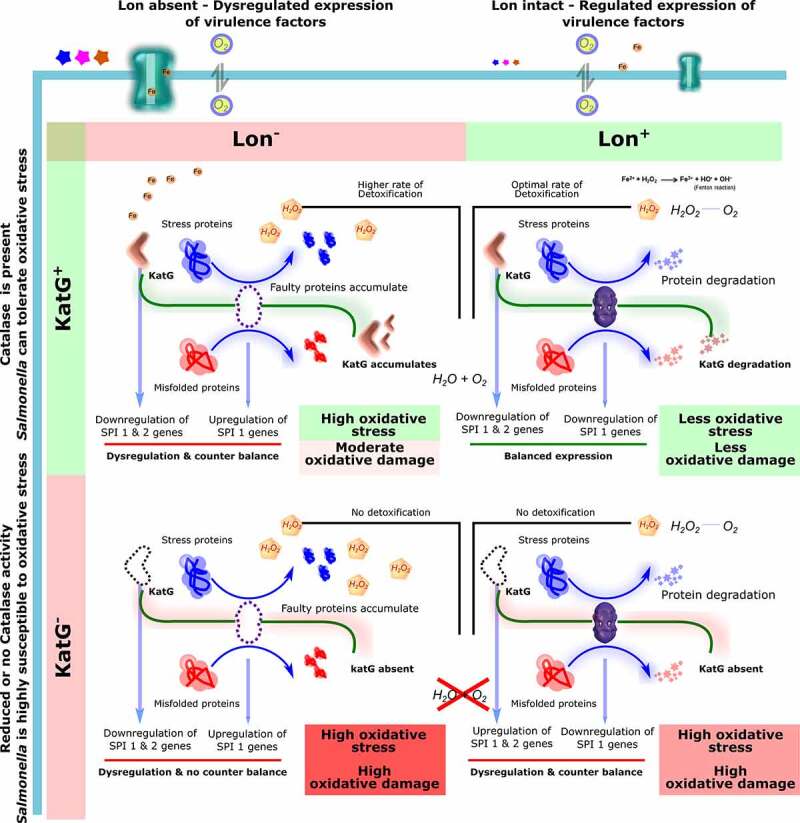
**Proposed interaction mechanism of Lon and KatG in *Salmonella***. The expression of KatG is potentially regulated by Lon protease. When both Lon and KatG are intact, *Salmonella’*s virulence and oxidative stress tolerance are ideally maintained for maximum intracellular survival. When *lon* is deleted, overexpressed virulence factors enhance the oxidative damage; to mitigate the stress imparted by the host cell, levels of KatG increases in the bacteria for the efficient detoxification of H_2_O_2_. Hydrogen peroxide is converted into H_2_O and molecular oxygen, relieving the harmful effect of ROS on cellular components. When both *lon* and *katG* are eliminated, the bacterium is extremely susceptible to ROS due to the absence of an oxidative stress response mechanism.

The present study demonstrates the ability of proteome analysis to discover the role of global regulator Lon protease in regulating protein expression. Our observation that *katG*, a gene encoding catalase-peroxidase is controlled by Lon protease is further evidence that is involved in regulating stress response by controlling the production of other bacterial antioxidants. Further, our study showed that *katG and lon* are essential to combat exogenous and endogenous ROS. ST survival and proliferation in macrophages is regulated by *katG*. Although SPI-2 effectors are regulated differently by *lon* and *katG*, SPI-1 is positively regulated by these enzymes. Based on the collective findings (Figure 8), it can be concluded that both *lon* and *katG* contribute to robust oxidative stress defense mechanism and virulence of ST and that the simultaneous loss is detrimental to the virulence of the bacterium.

## Materials and Methods

### Ethics statement

Specific pathogen-free female mice aged five weeks were obtained from Koatech in Pyeongtaek, Korea. All animals were housed in a temperature- and humidity-controlled room, in which a 12-h light/12-h dark cycle was maintained. Experiments with animals were approved by the Jeonbuk National University Animal Ethics Committee (CBNU2015-00085) following the guidelines of the Korean Council on Animal Care and Korean Animal Protection Law, Article 13 (2007).

## Bacterial strains, plasmids, and culture conditions

The bacterial strains and plasmids used in this study are listed in Table 2. All mutant *Salmonella* strains were derived from WT *Salmonella* Typhimurium strain JOL401 using the lambda red recombination method.^50^ *Salmonella* strains were routinely grown in Luria Bertani (LB; BD, USA) at 37°C with or without agitation. High levels of aeration were achieved by culturing in Erlenmeyer flasks subjected to vigorous shaking. To enhance aeration, we reduced the culture volume from 10 ml to 4 ml to increase headspace and increased tilt angle during shaking at 220 rpm. In contrast, low oxygen levels were maintained using cultures grown in screw cap tubes without agitation. Ampicillin, chloramphenicol, and kanamycin were used at 100 μg/ml, 25 μg/ml, and 50 μg/ml, respectively. Arabinose and IPTG (isopropyl-β-d-thiogalactopyranoside) were used to induce the gene expression from the plasmid constructs.

**Table 2.** List of bacterial strains and plasmids used in this study

Bacteria/ Plasmid	Genotypic characteristics	Reference
*S*.Typhimurium		
JOL 401	*Salmonella* Typhimurium wild type, SPI-1 *invAE^+^hilA^+^avr^+^*; SPI-2, amino acid permease; SPI-3, *mgtC^+^*; SPI4, ABC transporter; SPI5, *pipB^+^*	Lab stock
JOL909	JOL 401 ∆*lon*	Lab stock
JOL2469	JOL 401 ∆*katG*	This study
JOL2469::*katG*	JOL 2469 carrying pWSK29+ *katG* gene	This study
JOL2506	JOL 2469 ∆*clpP* (∆*katG*∆*clpP*)	This study
JOL2508	JOL 2469 ∆*ftsH* (∆*katG*∆*ftsh*)	This study
JOL2510	JOL 2469 ∆*hsIUV* (∆*katG*∆*haIUV*)	This study
JOL2755	JOL 909 ∆*katG* (∆*katG*∆*lon*)	This study
JOL2823	JOL 2755 carrying pWSK29+ *katG* gene	This study
*E. coli*		
DH5α	*E .coli* F^−^Φ80d*lac*ZΔM15Δ (*lac*ZYA-*arg*F)U169 *rec*A1 *end*A1 *hsd*R17(r_k_^−^, m_k_^+^) *pho*A *sup*E44 *thi*-1 *gyr* A96 *rel*A1λ^−^	Invitrogen
BTH101	F-cya-854, recA1, endA1, gyrA96 (Nalr),thi1, hsdR17, spoT1, rfbD1, glnV44(AS)	N/A
JOL2558	*KatG*::pET28(a) in DH5α	This study
JOL2583	*Lon PD*::pET28(a) in DH5α	This study
JOL2799	pUT18*-katG* in DH5α	This study
JOL2800	pKT25-*lon PD* in DH5α	This study
JOL2802	pUT18-*katG*(1–206) in DH5α	This study
JOL2803	pUT18-*katG*(1–207) in DH5α	This study
JOL2804	pUT18-*katG*(1–208) in DH5α	This study
JOL2805	pUT18-*katG*(1–209) in DH5α	This study
JOL2806	pUT18-*katG*(1–210) in DH5α	This study
JOL2807	pUT18-*katG*(1–211) in DH5α	This study
JOL2808	pUT18-*katG*(1–379) in DH5α	This study
JOL2809	pUT18-*katG*(1–380) in DH5α	This study
JOL2810	pUT18-*katG*(1–381) in DH5α	This study
JOL2811	pUT18-*katG*(1–382) in DH5α	This study
JOL2812	pUT18-*katG*(1–383) in DH5α	This study
JOL2813	pUT18-*katG*(1–384) in DH5α	This study
Plasmid		
pWSK29	Low copy cloning vector, Amp^R^	Lab stock
pKD46	oriR101-repA101ts; encodes lambda red genes (exo, bet, gam); native terminator (tL3); arabinose-inducible promoter for expression (ParaB); bla, Amp^R^	Lab stock
pKD3	oriR6Kgamma, bla (ampR), rgnB (Ter), cat^R^, FRT	Lab stock
pBAD	L-arabinose inducible, araBAD promoter, pBR322 Amp^R^	Lab stock
pUT18	P*lac* ColEI*ori* Amp^R^	N/A
pKT25	P*lac* ColEI*ori* Km^R^	N/A

## Construction of gene deletion *Salmonella* mutants

The genes encoding KatG, Lon, ClpP, ftsH, and hsIUV were deleted from the genome of *Salmonella* Typhimurium following the previously described protocols.^7,50^ The primers (cat^R^) used in the gene deletion are listed in Table 2. The successful gene deletion was screened by using primers pairs for the flanking regions (flanking primers) and coding sequence of the gene (inner primers).

## Plasmid construction

The *katG* open reading frame (ORF) from *Salmonella* Typhimurium was amplified by PCR and cloned into a pBAD expression vector using *EcoR1* and *HindIII* restriction enzymes. The respective primer pairs used in amplification are described in Table 3.

**Table 3.** List of primers used in this study

Gene	Sequence (5’-3’)	Reference
*katG ^catR^*	F:ACTTCCCGTTGCCAGCGCCTCTTTCATTATAACCCTGTGTTTATTATGAAGTGTAGGCTGGAGCTGCTTC R:AGCATCGTTTTTGCCAATTCCCTCCCCGAATGAGGGAGGGAAGGTTGCCAATGGGAATTAGCCATGGTCC	This study
*katG*-Flanking	F: ACTTCCCGTTGCCAGCGCCTCTTTC R: AGCATCGTTTTTGCCAATTCCCTCC	This study
*katG-*Inner	F: GACGGTAAACCCGTCACCAG R: ACTCCCGGTACGTTCAGACT	This study
*clpP ^catR^*	F:TTGAGGGATGACCTCATTTAATCTCCAGTAGCAATTTTGACCTGTTATGGGTGTAGGCTGGAGCTGCTTC R:TTTTAAACAAAAACGAGCCCGTCAGGGCCCGTTTTATTCAAATTTGTGACATGGGAATTAGCCATGGTCC	This study
*clpP*- Flanking	F: ATTACCACCACCGCATACC R: GCTTCGACTATCTCAGCTAAC	This study
*clpP*-Inner	F: TCAATAAATCACATCCCAAGCC R: CCCTTCAGCAAAATTAAGCATC:	This study
*ftsH ^catR^*	F:TGACGCTGTTTTTAACACAGTTGTAATAAGAGGTTAATCCCTTGAGTGACGTGTAGGCTGGAGCTGCTTC R:AAGCCCCAGGGTTTCGGTGAGCGCTAAACATAATGTTGTAAAACAAATGCATGGGAATTAGCCATGGTCC	This study
*FtsH*- Flanking	F: GCGAAAGTGATAACCGGCAG R: CGGATGAGTGAGATCGAGCG	This study
*FtsH*- Inner	F: GGGTTGTGACGAAGCGAAAG R: AAGCCGTCCATCTCAACCAG	This study
*hsIUV ^catR^*	F:TGCATTATGCCCCGTACGCCGTACGGGGCCGCAATTCAGCATTAGTAACCGTGTAGGCTGGAGCTGCTTC R:TTCAGTTCGCTGACAATTTCGCGTGGGGTCATTTCAGACATGAGAGGTCCATGGGAATTAGCCATGGTCC	This study
*hsIUV*-Flanking	F: AAGATGGCGGGCCATACAAA R: CATCAAGCTGCATACGACGC	This study
*hsIUV* – Inner	F: GGCAACGTGAAGAAAGTCCG R: GCCTGCAATATCCAACGCCT	This study
*KatG (pET28)*	F: GAATTCATGAGCACGACCGACGATAC R: AAGCTTGCTGCCGTCAAAGTTGGTTC	This study
*LonPD (pET28)*	F: GCGAATTCGAAAACCGCGTAGGT R: GGAAGCTTGTTTTGCAGAGCAAG	This study
***B2H primers***		
*katG*	F: CGGGATCCCATGAGCACGACCGACGAT R: CGGGTACCGCTTGCAGATCGAAACGGTC	This study
*Lon PD*	F: CGGGATCCCGAAAACCGCGTAGGTCAG R: CGGGTACCGCGTTTTGCAGAGCAAGCGT	This study
*katG* (1–206)*	F: CGGGATCCCATGAGCACGACCGACGAT R: CGGGTACCGCGTGAGTCAACCAGGCTTT	This study
*katG* (1–207)	F: CGGGATCCCATGAGCACGACCGACGAT R: CGGGTACCGCTCGGTGAGTCAACCAGGCT	This study
*katG* (1–208)	F: CGGGATCCCATGAGCACGACCGACGAT R: CGGGTACCGCGTGTCGGTGAGTCAACCA	This study
*katG* (1–209)	F: CGGGATCCCATGAGCACGACCGACGAT R: CGGGTACCGCAGGGTGTCGGTGAGTCAAC	This study
*katG* (1–210)	F: CGGGATCCCATGAGCACGACCGACGAT R: CGGGTACCGCTTCAGGGTGTCGGTGAGTC	This study
*katG* (1–211)	F: CGGGATCCCATGAGCACGACCGACGAT R: CGGGTACCGCCGCTTCAGGGTGTCGGTGA	This study
*katG* (1–379)	F: CGGGATCCCATGAGCACGACCGACGAT R: CGGGTACCGCCGTCAGGTCGGTGACCAG	This study
*katG* (1–380)	F: CGGGATCCCATGAGCACGACCGACGAT R: CGGGTACCGCCAGCGTCAGGTCGGTGAC	This study
*katG* (1–381)	F: CGGGATCCCATGAGCACGACCGACGAT R: CGGGTACCGCACGCAGCGTCAGGTCGGT	This study
*katG* (1–382)	F: CGGGATCCCATGAGCACGACCGACGAT R: CGGGTACCGCAAAACGCAGCGTCAGGTC	This study
*katG* (1–383)	F: CGGGATCCCATGAGCACGACCGACGAT R: CGGGTACCGCATCAAAACGCAGCGTCAG	This study
*katG* (1–384)	F: CGGGATCCCATGAGCACGACCGACGAT R: CGGGTACCGCCGGATCAAAACGCAGCGT	This study
***qRT-PCR***		
***Salmonella***		
*rrsG*	F: GTTACCCGCAGAAGAAGCAC R: CACATCCGACTTGACAGACC	This study
*sopE*	F: TTCAAGCCCCACTTCAAGCC R: TGAGTCAAATCCAGAACATGCC	This study
*sseJ*	F: ACGGATGCGACAAAAATCAC R: ACCTTGGAAGCCCTACAGAC	This study
*sifA*	F: AGACAATGAACGCTACACAC R: TCTTTTCTTCCACATCTATGCC	This study
*invC*	F: GACAACATACTGCTAACCGAC R:ATCACTCTTCACCTGCTCC	This study
*katE*	F: ATTCCGGAAGAGTTGGTGCCAGTA R: GACGACTGATTTGCGTGTCGGTAT	^51^
*katN*	F: GCGCGAGCGAAGATCATTTAT R: GCGACTTCACGGGTCATTAAGA	^51^
*katG*	F: TTAACTCCTGGCCGGATAAC R: TAATCGGCCACAACAAACG	^51^
*TsaA/ahpC*	F: TTTGTTTGCCCGACTGAACTGG R: TGTGCGTGAAGTGAGTATCGGT	^51^
*ahpF*	F: GGCTATCGATCTGGCAGGTATTGT R: TACGCACTTTGTCCTGTAGCA	^51^
**Mouse**		
*IL-2*	F: CCTGAGCAGGATGGAGAATTACA R: TCCAGAACATGCCGCAGAG	^52^
*IL-6*	F: GAGGATACCACTCCCAACAGACC R: AAGTGCATCATCGTTGTTCATACA	^52^
*TNF-α*	F: CATCTTCTCAAAATTCGAGTGACAA R: TGGGAGTAGACAAGGTACAACCC	^52^
*GAPDH*	F: TCACCACCATGGAGAAGGC R: GCTAAGCAGTTGGTGGTGCA	^52^
**Human**		
*IL-2*	F: AACTCACCAGGATGCTCACATTTA R: TCCCTGGGTCTTAAGTGAAAGTTT	^52^
*IL-6*	F: CATCCTCGACGGCATCTCAG R: GCTCTGTTGCCTGGTCCTC	^53^
*TNF-α*	F: TCTTCTCGAACCCCGAGTGA R: CCTCTGATGGCACCACCAG	^52^
*GAPDH*	F: GGAAGGTGAAGGTCGGAGTC R: CAGCCTTGACGGTGCCATG	^53^

F-Forward primer; R- Reverse primer. *The numbers in parenthesis indicate corresponding amino acid residues.

## 2-D gel electrophoresis and analysis

Proteome isolation was conducted in *Salmonella* wild-type strain JOL401 and Lon protease mutant strain JOL909. Overnight cultures of both strains were freshly inoculated into LB broth (2% inoculum) and allowed to grow until the late log phase. When the OD_600_ reached 0.8–1.0, cells were harvested by centrifugation at 13000 × g for 30 min. Cells were washed with phosphate-buffered saline (PBS) and subjected to osmotic lysis using the hypotonic solution with 1% lysozyme. Purified proteins were quantified using the Bradford method. 2-D gel electrophoresis was conducted as described in a previous report.^54^ Resolved gels were stained with Coomassie blue. Proteins of interest were further characterized using amino-acid N-terminus sequencing after blotting onto a polyvinylidene difluoride membrane. The obtained sequences were used as a query in a BLAST analysis of the ST genome through the National Center for Biotechnology Information.^55^

## Immunoblot analysis

The expression of *katG* in ST strains was confirmed by western blot analysis. Strains were grown to 0.6 OD at OD_600_. Four-milliliter cell samples were adjusted to have the same concentration (1 × 10^8^ cells/ml), and all cells were collected by centrifugation. Cells were washed with PBS once and suspended in 0.5 ml of PBS. Cell lysis was carried out under denatured conditions using 8 M urea and brief sonication. The whole-cell lysate was filtered, and 20 µl of lysate was resolved in 12% SDS PAGE and transferred to a polyvinylidene difluoride membrane. Membranes were blocked with 5% BSA and incubated with anti-KatG polyclonal antibodies at 1:500 dilution. After two hours of incubation at 37°C, membranes were washed four times with 0.01% PBS-T and incubated with HRP-tagged anti-rabbit IgG as the secondary antibody (Southern Biotech, USA). Color development was achieved by adding 3, 3’-diaminobenzidine (DAB; Sigma Aldrich, USA) substrate.

## Bacterial two-hybrid assay

Potential interactions between Lon protease and KatG were evaluated using the bacterial two-hybrid (B2H) assay.^56,57^ The *E. coli* BTH101 (cyaA) strain was co-transformed with derivatives of the pUT18 and pKT25 plasmids. The *E. coli* host and plasmids for the B2H assay were a kind gift from Professor Eun-Jin Lee, School of Life Sciences and Biotechnology, Korea University. Transformants were grown in LB medium supplemented with 100 μg/ml ampicillin and 50 μg/ml kanamycin, at 37°C overnight. Then, 2 μl from broth cultures were withdrawn and spotted on solid MacConkey-maltose agar containing 100 μM IPTG, 100 μg/ml ampicillin, and 50 μg/ml kanamycin. Plates were incubated at 30°C for 40 h. In addition, the results were validated by quantitative β-galactosidase assay, as described in a previous study.^58^ Bacteria co-transformed with empty or zip vectors served as negative and positive controls, respectively.

## Adhesion and invasion assays

The adhesion and invasion potentials of the *Salmonella* mutants were evaluated in mice macrophage cell line RAW 264.7.^7^ Macrophage cells were cultured in 24-well plates and subjected to infection at a multiplicity of infection (MOI) of 20. After 45 min of incubation, cells were washed three times with PBS. Cell lysis was conducted by adding 1 ml of PBS with 0.1% Triton X-100 to each well for 10 min. Lysed cells were collected, and 100 μl was pipetted out and plated on LB agar for colony counting. Regarding invasion assays, bacteria were allowed to interact with the cell monolayer for 2 h and were subsequently treated with gentamycin (100 μg/ml) for 1.5 h to remove any extracellular bacteria. Then, the cells were lysed with 0.1% Triton X-100 for 10 min. Invaded cells were enumerated by plating on LB agar using decimal dilutions. The data from three independent experiments with standard deviation are presented as CFU/mL.

## Macrophage survival assay

ST mutant macrophage survival was conducted in mice macrophage cell line RAW264.7. Cells were seeded in a 24-well plate at a density of 5 Χ 10^5^ cells per well in Dulbecco’s modified Eagle’s medium (DMEM; Lonza, Switzerland) supplemented with 10% fetal bovine serum (FBS; Serana, Germany) and cultured at 37°C and 5% CO_2_ in a humidified atmosphere. At confluence, Bacteria grown to mid-log phase were added onto macrophage cells at 0.1 MOI. Plates were centrifuged at 1000 rpm for 5 min at room temperature and continued to incubate for 20 min. Extracellular bacteria were eliminated by washing with PBS three times. Then wells were replenished by complete medium, DMEM, 10% FBS containing 120 μg/ml gentamycin, and incubated for 1 h. Bacterial survival was measured at 1- and 18-h time intervals by cell lysis after adding PBS and 0.1% Triton X-100 as mentioned earlier. Percent survival was calculated for the 18-h time point in relation to the number of bacteria recovered at the 1-h time point. This experiment was conducted in triplicate.

## Hydrogen peroxide sensitivity assay

The effect of H_2_O_2_ on the growth of ST wild-type and mutants was evaluated using a previously described protocol.^59^ Briefly, a triplicate of overnight bacterial cultures were diluted to 1:1000 into 10 ml LB in 50 ml conical tubes and were grown to the mid-exponential phase. Cultures were treated with various concentrations of H_2_O_2_ (1, 2, and 4 mM, prepared from 30% H_2_O_2,_ 9.8 M). The oxidative stress response was evaluated at 3 and 6 hours after H_2_O_2_ treatment. At each time point, 10 μl of culture was collected, serially diluted, and plated onto LB agar supplemented with catalase (Sigma Aldrich, USA) for CFU enumeration. Furthermore, a disk diffusion assay was performed to test H_2_O_2_ sensitivity, as described previously.^60^ The ST strains were cultured under near-anaerobic conditions to the mid-log phase, and 100 μl aliquots were spread on LB plates. A sterile 5-mm diameter filter disk (Sigma Aldrich, USA) containing 4 μl of 2 or 4 mM H_2_O_2_ was placed on the surface of the ST-containing LB plate. After incubation at 37°C for 12 h, the size of the area cleared of bacteria (inhibition zone) was measured using a Vernier caliper.

## ROS detection in bacteria

The role of *lon* and *katG* in the accumulation of ROS in the ST strains was determined by analyzing the detection of 2,7-dichlorofluorescein diacetate (DCFH2-DA) fluorescence (Sigma Aldrich, USA). A stock solution of 10 mM (w/v) DCFH-DA in DMSO was prepared and kept at −20°C in the dark for further use. A 10 μM working solution was prepared in PBS. The bacteria grown to mid-log growth were collected and washed 3 times with PBS buffer to detect the production of ROS. First, 10 μM of DCFH-DA was added to the bacterial cultures and incubated on a shaker at room temperature in the dark for 30 minutes. Then, the bacterial culture was placed on a glass slide with a coverslip and visualized under a fluorescent microscope with a filter for fluorescein isothiocyanate (FITC) (Zeiss, Germany). Additionally, the production of DCF was measured immediately on a fluorescent plate reader at 485-nm excitation and 535-nm emission in the endpoint mode.

## Quantification of catalase activities

The catalase activity in the wild-type and the mutant strains was quantified using a previously published method.^61^ Briefly, bacterial suspension of 1 × 10^8^ CFUs in 100 μL volume was added in a Pyrex tube (13 mm diameter × 100 mm height, borosilicate glass; Corning, USA). Subsequently, 100 μL of 1% Triton X-100 and 100 μL of 30% hydrogen peroxide were added to the solutions and mixed thoroughly, and the solutions were then incubated at room temperature for 15 min. No reagents were used to stop the reaction, as the generation of oxygen stops naturally within 5 min. Following completion of the reaction, the height of O_2_-forming foam, which remained constant for 15 min, in the test tube was finally measured using a Vernier caliper.

## qRT-PCR for the evaluation of gene expression

Cytokine gene expression in Caco-2 and RAW 294.7 cells in response to ST infection was quantified, with IL-2, IL-6, and TNF-α having been investigated. Caco2 and RAW 294.7 cells were infected with ST WT and mutant strains at an MOI of 20 and incubated for 12, 24, and 48 h. Cells were harvested at each time interval, and the total RNA was isolated; the corresponding cDNA was then synthesized (Elpis Biotech, Korea). The expression levels of cytokine genes were quantitatively analyzed using qRT-PCR (Table 3). The expression of cytokine genes was normalized against *GAPDH* expression, and the changes in the relative expression of cytokine genes were determined using the 2^−ΔΔCT^ method.^62^ The mRNA levels of ST virulence-associated genes were compared in the wild-type and mutant strains. The SPI 1 and SPI 2 gene expression was analyzed in *Salmonella* recovered from cells at 20 min and 3 h post-infection, respectively. The time was chosen to specifically reflect the activation of SPI 1 and SPI 2 genes as they play a role in cell adhesion and intracellular survival, respectively. The expression levels of bacterial genes were normalized against the ST *rrsG* housekeeping gene.

## Mouse virulence assay

Six-week-old female BALB/c mice (n = 10/group) were inoculated with ST strains at 1 × 10^5^ CFU/mouse/100 µl PBS. Mice were monitored daily for ST infection and associated clinical signs. On days 1, 3, 6, 9, and 12 post-infection, mice were euthanized, and the spleens were aseptically collected. Whole spleens were homogenized in 3 ml PBS using a mechanical homogenizer (IKA T 10 basic ULTRA-TURRAX, Germany). A hundred microliters of homogenate were serially diluted and plated on Brilliant Green Agar (BGA) for bacterial enumeration. Furthermore, the bacterial colonization associated with tissue damage in the spleen was assessed by H&E staining according to standard protocols.

## Statistical analysis

Statistical analysis was conducted using GraphPad Prism 7 (GraphPad Software, CA, USA) and IBM SPSS software. Student’s t-test and analysis of variance (ANOVA) followed by Turkey’s multiple comparison test was used to compare means among the treatment groups and to compute the corresponding p-values. P-values ≤0.05 were considered to demonstrate a statistically significant comparison.

## Supplementary Material

Supplemental Material

## Data Availability

The authors confirm that the data supporting the findings of this study are available within the article and/or its supplementary materials.
