# Supplementary material for: Coordinated interaction between Lon protease and catalase-peroxidase regulates virulence and oxidative stress management during Salmonellosis
Source: Gut Microbes. 2022 Apr 19;14(1):2064705. doi: 10.1080/19490976.2022.2064705 (PMC9037549; doi:10.1080/19490976.2022.2064705)
Supplement: Supplemental Material [file KGMI_A_2064705_SM2664.doc]

**Prediction of KatG cleavage sites for Lon PD using PROSPER**

**KatG (Catalase-peroxidase)**

**The predicted cleavage sites**

MSTTDDTHNTLSTGKCPF**/**HQGGHDRSAGAGTASRDWW**/**PNQLRVDLLNQHSNRSNPLGEDFDYRKEFSKLD

YSALKGDLKALLTDSQPWWPADWGSYVGLFIRMAW**/**HGAGTY**/**RSIDGRGGAGRGQQRFAPLNSWPDNVSLD

KARRLLWPIKQKYGQKISWADLFILAGNVALENSGFRTFGFGAGREDVWEPDLDVNWGDEKAWLTHR**/**HPE

ALAKAPLGATEMGLIYVNPEGPDHSGEPLSAAAAIRATFGNMGMNDEETVALIAGGHTLGKTHGAAAASH

VGADPEAAPIEAQGLGWASSYGSGVGADAITSGLEVVWTQTPTQWSNYFFENLFKYEWVQTRSPAGAIQF

EAVDAPDIIPDPFDPSKKRKPTMLVTDLTLR**/**FDPEFEKISRRFLNDPQAFNEAFARAW**/**FKLTHRDMGPKA

RYIGPEVPKEDLIWQDPLPQPLYQPTQEDIINLKAAIAASGLSISEMVSVAW**/**ASASTFRGGDKRGGANGA

RLALAPQRDWDVNAVAARVLPVLEEIQKTTNKASLADIIVLAGVVGIEQAAAAAGVSISVPFAPGRVDAR

QDQTDIEMFSLLEPIADGFRNYRARLDVSTTESLLIDKAQQLTLTAPEMTVLVGGMRVLGTNFDGSQNGV

FTDRPGVLSTDFFANLLDMRYEWKPTDDANELFEGRDRLTGEVKYTATRADLVFGSNSVLRALAEVYACS

DAHEKFVKDFVAAWVKVMNLDRFDLQ

**/- signalase**

**/- chymotrypsin A (cattle-type)**


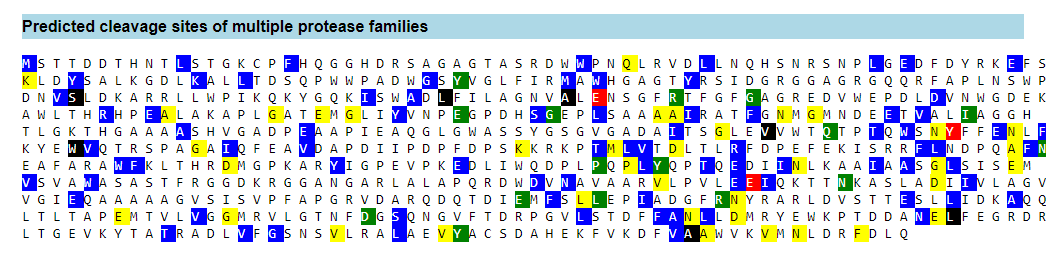


**Lon protease**

MVWSTAILSDYLADTKLRESSMNPERSERIEIPVLPLRDVVVYPHMVIPLFVGREKSIRCLEAAMDHDKK

IMLVAQKEASTDEPGVNDLFTVGTVASILQMLKLPDGTVKVLVEGLQRARISALSDNGEHFSAKAEYLDS

PAIDEREQEVLVRTAISQFEGYIKLNKKIPPEVLTSLNSIDDPARLADTIAAHMPLKLADKQSVLEMSDV

NERLEYLMAMMESEIDLLQVEKRIRNRVKKQMEKSQREYYLNEQMKAIQKELGEMDDAPDENEALKRKID

AAKMPKEAKEKAEAELQKLKMMSPMSAEATVVRGYIDWMVQVPWNARSKVKKDLRQAQEILDTDHYGLER

VKDRILEYLAVQSRVNKIKGPILCLVGPPGVGKTSLGQSIAKATGRKYIRMALGGVRDEAEIRGHRRTYI

GSMPGKLIQKMAKVGVKNPLFLLDEIDKMSSDMRGDPASALLEVLDPEQNVAFSDHYLEVDYDLSDVMFV

ATSNSMNIPAPLLDRMEVIRLSGYTEDEKLNIAKRHLLPKQIERNALKKGELTVDDSAIIGIIRYYTREA

GVRSLEREISKLCRKAVKQLLLDKSLKHIE**I**NGDNLHDYLGVQRFDYGRADSENRVGQVTGLAWTEVGGD

LLTIETACVPGKGKLTYTGSLGEVMQESIQAALTVVRARAEKLGINPDFYEKRDIHVHVPEGATPKDGPS

AGIAMCTALVSCLTGNPVRADVAMTGEITLRGQVLPIGGLKEKLLAAHRGGIKTVLIPFENKRDLEEIPD

NVIADLDIHPVKRIEEVLTLA**L**QNEPSGMQVVTAK

Region 22..805 (region name="PRK10787")

DNA-binding ATP-dependent protease La

Region 344..>393 (region name="P-loop_NTPase")

P-loop containing Nucleoside Triphosphate Hydrolases

Region 591..792 (region name="Lon_C")

Lon protease (S16) C-terminal proteolytic domain

**I**NGDNLHDYLGVQRFDYGRADSENRVGQVTGLAWTEVGGD

LLTIETACVPGKGKLTYTGSLGEVMQESIQAALTVVRARAEKLGINPDFYEKRDIHVHVPEGATPKDGPS

AGIAMCTALVSCLTGNPVRADVAMTGEITLRGQVLPIGGLKEKLLAAHRGGIKTVLIPFENKRDLEEIPD

NVIADLDIHPVKRIEEVLTLA**L**


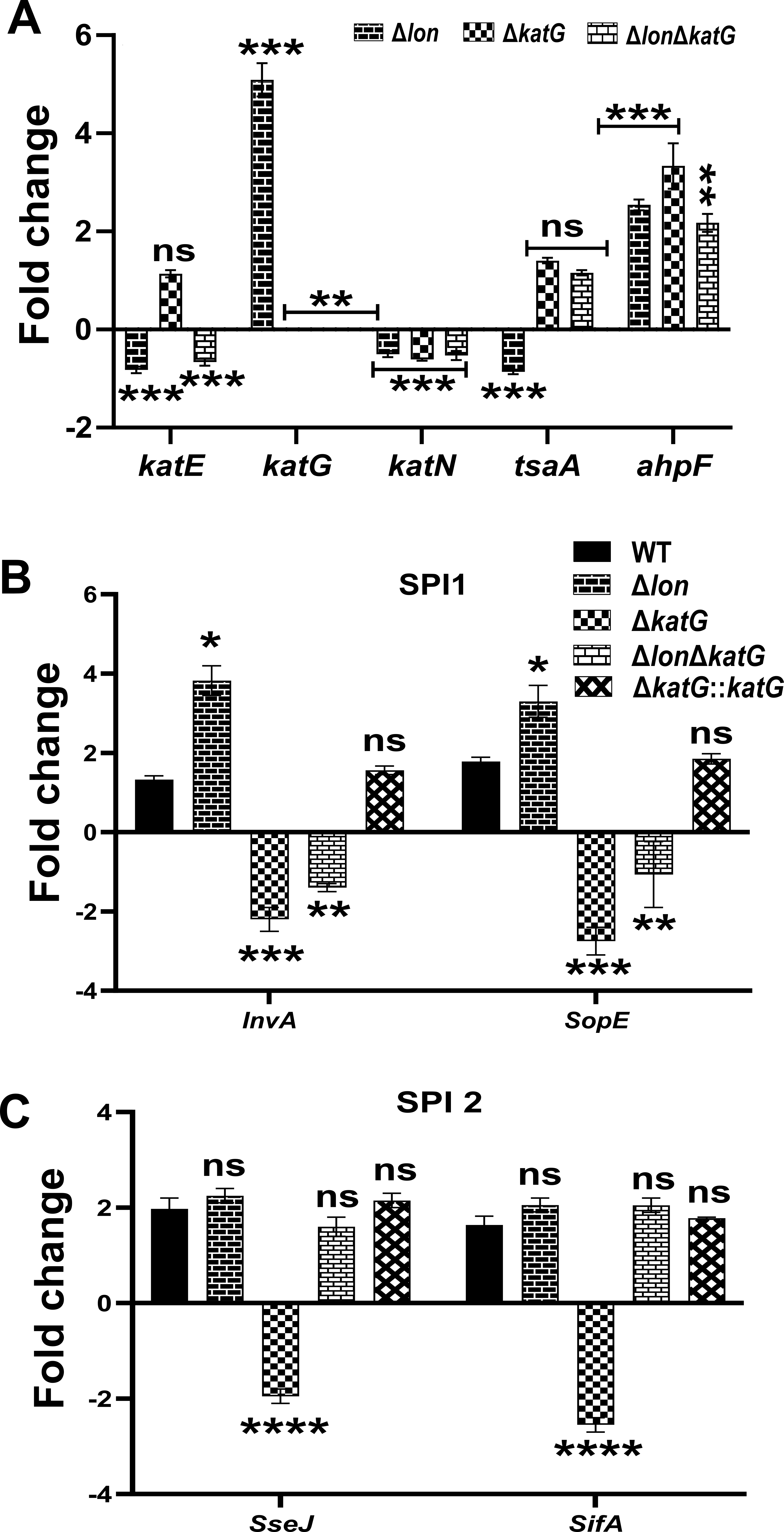


**Supplementary Figure 1:** Assessment of gene expression in ST strains. Total RNA was extracted from the ST strains in the log phase of growth and cDNA was prepared. (A) Graph depicting the expression of *katE, katG, katN, ahpC* and *TsaA.* Graphs depicting the mRNA expression of SPI1 (B) and SPI 2 (C). Data was analyzed by ANOVA using Tukey's multiple comparisons test. (^ns^non-significant, *p < 0.05, **p < 0.01, ***p < 0.001, ****p < 0.0001).
